# Editorial: Highlights in gynecology 2021/22

**DOI:** 10.3389/frph.2022.1123881

**Published:** 2023-01-11

**Authors:** Laura Buggio, Nilufer Rahmioglu

**Affiliations:** ^1^ Gynecology Unit, Fondazione IRCCS Ca’ Granda Ospedale Maggiore Policlinico, Milan, Italy; ^2^ Oxford Endometriosis CaRe Centre, Nuffield Department of Women's and Reproductive Health, University of Oxford, Oxford, United Kingdom; ^3^ Wellcome Centre for Human Genetics, University of Oxford, Oxford, United Kingdom

**Keywords:** endometriosis, PCOS (polycystic ovarian syndrome), uterine fibroids, adenomyosis, gynecology

**Editorial on the Research Topic** Highlights in gynecology 2021/22

In this Frontiers in Reproductive Health Research Topic entitled “Highlights in Gynecology 2021/2022”, we present a collection of papers focusing on global themes central to women's health. This collection aims to highlight the broad diversity of research performed across the Gynecology section and to spotlight the main areas of interest. In addition, this Editorial introduces three manuscripts published as a collection in response to the above-mentioned Research Topic.

- Summary of “*Prevalence of Common Gynecological Conditions in the Middle East: Systematic Review and Meta-Analysis*” (Mousa et al.)

This systematic review critically assesses the evidence from epidemiological research and performs a meta-analysis of studies estimating the prevalence of endometriosis, polycystic ovary syndrome (PCOS), uterine fibroids, and adenomyosis in Middle Eastern populations. Most studies from the region have low sample sizes and there are inconsistencies in terms of the methodology and representativeness of the study samples; therefore, a meta-analysis of the available evidence from these studies represents a valuable contribution to the literature. The authors conclude that the pooled prevalence of PCOS diagnosed according to NIH criteria is 8.9% (range, 4%–27.6%), with the highest prevalence (18.8%) observed in the Gulf Arab states (range, 12.1%–27.6%). The prevalence of endometriosis is limited to women undergoing laparoscopic surgery (12.9%; range, 4.2%–21%), and the prevalence of adenomyosis in women undergoing hysterectomy is 30.8% (range, 25.6%–37.7%). The prevalence of uterine fibroids is estimated to be 28.2% overall in the region (range, 18.5%–42.6%) and 57.1% among women with heavy menstrual bleeding. The authors highlight the potential health disparities in these populations and call for further research to understand the regional environmental and genetic risk factors for these conditions.

- Summary of “*Ibero-American Endometriosis Patient Phenome: Demographics, Obstetric-Gynecologic Traits, and Symptomatology*” (Flores-Caldera et al*.*)

In this cross-sectional study, the authors collect self-reported data on the demographics, lifestyle, and endometriosis symptoms of Hispanic/Latinx endometriosis patients from April 2019 to February 2020. In general, Hispanic/Latinx individuals with endometriosis are underrepresented in research databases and are thus unlikely to benefit from any advancements in pathophysiology, biomarkers, and novel treatments. To fill this knowledge gap, Flores-Caldera et al. for the first time in the literature, define the clinical profile of endometriosis patients from Latin America and the Caribbean using a Spanish translation of EPHect's Minimal Clinical Questionnaire (EPQ-M). A total of 1,378 individuals from 23 countries answered the questionnaire. Patients were recruited through collaborations with patient associations in South America, Central America, the Caribbean, and Spain. This study represents the first attempt to generate a clinical-demographic profile of Hispanic/Latinx endometriosis patients. The survey results suggest that, compared with other cohorts, Hispanic/Latinx endometriosis patients reported a higher prevalence and severity of dysmenorrhea and dyspareunia and high levels of pain catastrophizing.

- Summary of “*Case Report: Extrapelvic Endometriosis in the Medial Thigh*” (Pascoal et al.)

Pascoal et al*.* present the case of a 39-year-old nulliparous woman with a rare form of extrapelvic musculoskeletal endometriosis in the medial thigh. Extrapelvic musculoskeletal endometriosis is a rare entity with likely multifactorial pathogenesis. Interestingly, the patient had a history of pelvic fractures. The authors formulate a possible fascinating pathogenic theory for the insurgence of extrapelvic endometriosis based on the stem/progenitor cell theory and the role that musculoskeletal trauma may have in the development of this condition. In addition, the authors explore the various management options for this rare form of the disease and suggest a multidisciplinary approach to ensure optimal margins and minimize morbidity secondary to surgical resection and defect closure.

Both Mousa et al. and Flores-Caldera et al. present epidemiological insights into common gynecological conditions in underrepresented populations in women's health. These valuable contributions are important for highlighting potential health disparities and the need to expand the basis of gynecological research to diverse populations to better understand the population-specific environmental and genetic risk factors. Lastly, Pascoal et al. describe a rare form of endometriosis and highlight the importance of approaching this complex condition's diagnosis and treatment in a multidisciplinary approach.

